# Progeny-testing of full-sibs IBD in a SSC2 QTL region highlights epistatic interactions for fatness traits in pigs

**DOI:** 10.1186/1471-2156-12-92

**Published:** 2011-10-27

**Authors:** Flavie Tortereau, Marie-Pierre Sanchez, Katia Fève, Hélène Gilbert, Nathalie Iannuccelli, Yvon Billon, Denis Milan, Jean-Pierre Bidanel, Juliette Riquet

**Affiliations:** 1 INRA, UMR444 Laboratoire de Génétique Cellulaire, 31326 Castanet-Tolosan, France; 2 Wageningen University, Animal Breeding and Genetics Group, 6700AH Wageningen, The Netherlands; 3 INRA, UMR1313 Génétique Animale et Biologie Intégrative, 78350 Jouy-en-Josas, France; 4 INRA, UE967 Génétique Expérimentale en Productions Animales, 17700 Surgères, France

## Abstract

**Background:**

Many QTL have been detected in pigs, but very few of them have been fine-mapped up to the causal mutation. On SSC2, the *IGF2*-intron3-G3072A mutation has been described as the causative polymorphism for a QTL underlying muscle mass and backfat deposition, but further studies have demonstrated that at least one additional QTL should segregate downstream of this mutation. A marker-assisted backcrossing design was set up in order to confirm the segregation of this second locus, reduce its confidence interval and better understand its mode of segregation.

**Results:**

Five recombinant full-sibs, with genotype G/G at the *IGF2 *mutation, were progeny-tested. Only two of them displayed significant QTL for fatness traits although four inherited the same paternal and maternal chromosomes, thus exhibiting the same haplotypic contrast in the QTL region. The hypothesis of an interaction with another region in the genome was proposed to explain these discrepancies and after a genome scan, four different regions were retained as potential interacting regions with the SSC2 QTL. A candidate interacting region on SSC13 was confirmed by the analysis of an F2 pedigree, and in the backcross pedigree one haplotype in this region was found to mask the SSC2 QTL effect.

**Conclusions:**

Assuming the hypothesis of interactions with other chromosomal regions, the QTL could be unambiguously mapped to a 30 cM region delimited by recombination points. The marker-assisted backcrossing design was successfully used to confirm the segregation of a QTL on SSC2 and, because full-sibs that inherited the same alleles from their two parents were analysed, the detection of epistatic interactions could be performed between alleles and not between breeds as usually done with the traditional Line-Cross model. Additional analyses of other recombinant sires should provide more information to further improve the fine-mapping of this locus, and confirm or deny the interaction identified between chromosomes 2 and 13.

## Background

Many QTL underlying economically important traits have been detected in pigs over the last fifteen years [1]. These QTL have usually been mapped in large intervals (10 - 30 cM) using experimental crosses between distant populations. Consequently, their use in pig selection schemes has been very limited so far. The only QTL fine-mapped up to the causal mutation in pigs is an A-G substitution in the third intron of the *IGF2 *gene (position 3072) [2]. This causative mutation influences muscle mass and backfat deposition in crosses between Large White (LW) and European Wild Boar [3], between LW and Pietrain [4] and between European breeds and Meishan (MS) [5]. For fat-related traits like backfat thickness, many QTL have been mapped on pig chromosome 2 (SSC2) within various experimental populations: their most likely positions ranging from 0 to 50 cM [6-9]. For crosses involving European breeds such as Piétrain, LW and Landrace, the *IGF2 *mutation effect was large enough to refrain from investigating further QTL affecting these traits. However, it has been demonstrated that this *IGF2 *mutation does not explain the whole genetic variation of the SSC2 QTL in a LW × MS cross [10], and studies of pedigrees where the *IGF2*-intron3-G3072A mutation was not segregating, indicated the presence of a fatness-related QTL between 30 and 60 cM [11].

In this study, we present QTL analyses in advanced backcross families produced after multiple directed crosses from a F2 LW × MS cross. These were produced from sires carrying recombinant LW × MS chromosomes with recombination points evenly distributed on SSC2p. This strategy, known as marker-assisted backcrossing, is usually performed to refine QTL mapping intervals [10,12,13]. The aims of this study were: 1) to confirm and fine-map the fatness-related QTL segregating between 30 and 70 cM on SSC2 and 2) to determine the mode of inheritance of this QTL using the F2 and advanced backcross populations.

## Methods

### Animals

Data analysed in this paper came from an advanced backcross population obtained by marker-assisted backcrossing and deriving from the French PorQTL F2 design described by Bidanel et al. [6]. The care and use of animals were performed in compliance with the guidelines of the French Ministry of Agriculture and Fisheries. Phenotypes of all the animals were recorded at the experimental farm and in a commercial abattoir in standard conditions.

The PorQTL pedigree was created by mating six Large White (LW) sires with six Meishan (MS) dams. Six F1 sires and twenty F1 dams were then mated to produce 1052 F2 animals. All pigs were born and raised at the INRA GEPA experimental unit (Poitou-Charentes). Semen from the F1 sires was frozen. Bidanel et al. [6] described QTL detection results obtained for major production traits from which QTL genotypes of the F1 LW × MS sires have been assessed.

**Figure 1:**
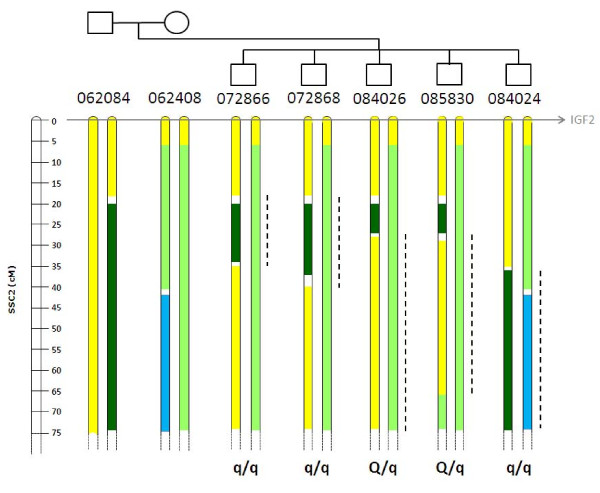
**SSC2 haplotypes of the five progeny-tested sires**. The pairs of haplotypes (0-75 cM) inherited by the five progeny tested sires from their two parents (062084 and 062408).Yellow refer to the IBD Meishan haplotype (associated with the backfat increasing QTL allele), all other colors refer to different Large White haplotypes (associated with the backfat decreasing QTL allele). In the white areas, the origin could not be defined. The progeny testing results obtained for each sire is indicated by a genotype at the SSC2 QTL (q/q for no segregating families, Q/q for segregating families). The dashed lines represent the position of the studied QTL based on the progeny-testing results of these sires.

The advanced backcross population was generated in the INRA GEPA experimental unit (Poitou-Charentes). To produce the first generation of males and females backcrosses (BC), LW dams were inseminated with previously frozen semen of a F1 LW/MS sire. The following five generations were dedicated to the introgression of the F1 sire MS haplotype in *IGF2 *region (from 0 to 7 cM) and to the production of various recombinant LW/MS segments in the SSC2 region located between 7 and 70 cM. After five generations of successive backcross, a panel of recombinant sires in the region 7 to 70 cM, homozygous MS/MS for the *IGF2*-intron3-G3072A mutation, was finally mated to LW sows and progeny-tested using on average 100 offspring per sire. In the present study, five half-sib families, obtained from five full-sib sires, were selected for fatness trait QTL analyses (Figure 1).

### Phenotypic Data

F2 animals from the PorQTL pedigree were assessed for average backfat thickness at 120 and 154 days of age. Details about those measurements can be found in [6]. In the present study, we assessed average backfat thickness at 120 days of age because this trait was measured in all F2 animals (n = 1071) in contrast to average backfat thickness at 154 days of age that was measured in females only (n = 542).

In the backcross population, all piglets were weaned at 28 days of age and were placed in postweaning collective pens until 10 weeks of age. They were then transferred to a fattening unit until 140 days of age. At 120 and 140 days of age, backfat thickness was measured using real-time ultrasound (Aloka SSD-500, Ecotron Aloka), on each side of the spine at 4 cm of the mid-dorsal line and 10 cm of the shoulder (neck), between the 3^rd ^and 4^th ^last ribs (back) and at the level of the last lumbar vertebra (rump). The pigs were finally slaughtered at a mean age of 175 ± 10 days in a commercial slaughterhouse (Saint-Maixent, Deux-Sèvres). Shortly after slaughter, carcass weights and lengths were recorded and carcass fat depths were measured at the shoulder, the last rib and the hip joint. Additional fat (G2) and lean (M2 and M6) depths were recorded between the 3^rd ^and 4^th ^last ribs at 6 cm off the mid-dorsal line using a Fat-o-Meat'er (SFK Technology A/S, Herlev, Denmark) probe. Sixteen traits related to fatness were defined from the above mentioned measurements and analysed: the mean of the two ultrasonic backfat thickness measurements at the level of the neck (**UBFn**), the back (**UBFb**) and the rump (**UBFr**) as well as the mean of the six measurements (**UBFm**) at 120 and 140 days of age; carcass fat depths at the level of the neck (**BFneck**), the back (**BFback**) and the rump (**BFrump**) and the mean of the three measurements (**BFmean**); fat depth **G2 **and lean depths **M2 **and **M6**; lean meat content (**LMC**) estimated using G2 and M2 measurements (LMC = 62.19 - 0.729 * G2 + 0.144 * M2).

### Genetic Data

Different sets of microsatellite markers were used for genotyping depending on the families. All amplifications were performed on ABI 9700 PCR machines (Applied Biosystems, Foster City, CA), and genotyping was carried out on an ABI 3730 automatic sequencer (Applied Biosystems). Genotypes were then determined using the Genemapper software (Applied Biosystems) and results of genotyping were checked, validated, and stored in the GEMMA database [14].

The F2 PorQTL population had already been genotyped for 123 microsatellites evenly spaced across the genome [6]. F0 and F1 animals were further genotyped for the *IGF2*-intron3-G3072A mutation, and the genotypes of the F2 at this mutation were inferred as explained in [15].

**Figure 2:**
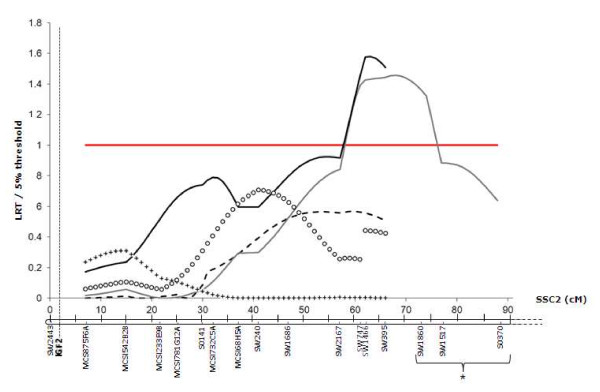
**LRT profile on SSC2 for live backfat thickness measured at the level of the neck at 140 days of age in the five sire families**. The crossed, circled, grey, black and dashed lines give the results for sires 072866, 072868, 084026, 085830, and 084024, respectively. The LRT is presented as a ratio to the 5% threshold on the linkage group. *SW1860, SW1517 and S0370 were not informative for the 085830, 084024, 084026, 072868 and 072866 sire families.

Each of the five progeny-tested families (sire and progeny) from the backcross pedigree was genotyped for a set of informative microsatellites covering the 7-70 cM region on SSC2 (marker names and positions are reported in Figure 2). An additional set of 578 markers covering all the autosomes and SSCX were genotyped on the four full-sib sires, from which eight markers on SSC1, SSC3, SSC6, SSC8, SSC13, SSC14 and SC16 were selected and genotyped on their progeny to test epistatic interactions.

### Statistical analysis

Phenotypic data of the backcross families were adjusted for fixed effects (sex and batch) and covariates (weight at measurement or carcass weight for ultrasonic backfat thickness and carcass composition traits, respectively) using the GLM procedure of SAS (SAS 9.1, SAS Institute, Inc.). Average backfat thickness measured at 120 days of age on PorQTL F2 animals was corrected for sex, batch, weight at measurement and their genotype at the IGF2 mutation.

#### - *QTL analyses*

QTL detection was performed for each backcross family on the adjusted data using the QTLMap software [16,17] as explained by Tortereau et al. [18], from 7 to 70 cM (or 7 to 90 cM for the sire 084026). Parameter estimates were obtained by likelihood maximization using a Newton-Raphson algorithm, and a Likelihood Ratio Test (LRT) was computed at each centimorgan along the linkage group. The maximum LRT indicated the most likely position for the QTL. For each sire, the substitution effect corresponds to the difference between its maternal and paternal chromosomes. In our case a positive effect indicates an increase of the trait value attributable to the maternal LW chromosome. Conversely, a negative significant effect indicates an increased trait value due to the paternally inherited MS chromosomal segment of the recombinant chromosome. QTL significance thresholds were empirically computed using 1,000 simulations under the null hypothesis, assuming an infinitesimal polygenic model for the trait, as described by Gilbert and Le Roy [19].

#### - *Epistatic interactions analyses*

Epistatic interactions were assessed on the backcross and F2 animals by testing whether the detection of the QTL on SSC2 was conditional on the segregation of another locus in the genome. Four backcross families (from sires 072866, 072868, 084026, 085830) were retained for this analysis, with sires being full-sibs that inherited the same duo of parental chromosomes in the tested region of SSC2 (37-67 cM). In the SSC2 region the sires thus shared pairs of identical by descent chromosomes. The four backcross families were first clustered into two groups of two families (k = 1,2) depending on the SSC2 QTL genotype estimate (q/q or Q/q). Epistatic interactions were then tested within each group, between the SSC2 chromosomal region and every other candidate position on the genome marked with microsatellites. First, each group of progeny was divided in sub-groups considering the inherited paternal allele at the tested microsatellite (from two to three sub-groups g with 36 to 93 progeny). Then, within each group k, the QTL detection in the SSC2 region (from 30 to 80 cM) was assessed with the following model:

(1) $$\text{per}{\text{f}}_{i}=\mu_{\text{g}}+{{\text{p}}_{\text{SSC2i}}}^{*}\alpha_{\text{SSC2(kg)}}+\varepsilon_{i}$$

where, for all progeny *i *belonging to sub-group g of group k, μ_g _is the trait mean for the group, α_SSC2(kg) _is the substitution effect of the putative QTL for the sub-group g, p_SSC2i _is the probability for individual *i *to inherit its sire maternal allele at the tested position on SSC2 and ε_i _is the residual error.

Epistatic interactions in the F2 pedigree were tested with an adaptation of model (1). Because QTL analyses had been previously performed on this design [6], breed origin (LW or MS) of the paternally transmitted allele in the candidate regions was known for each progeny, and could be used to define two groups of offspring. Within each of these two groups, model (1) was used, with p_SSC2i _being computed as the probability that the progeny inherited a MS allele from its sire at the tested position *i *on SSC2.

When necessary, epistatic interactions were also tested in the backcross families between the SSC2 chromosomal region and a candidate region traced using transmitted paternal haplotypes in the progeny. Model (1) was jointly applied on the four backcross families with the following modifications:

(2) $$\text{per}{\text{f}}_{i}=\mu_{\text{g}}+{{\text{p}}_{\text{SSC2}i}}^{*}\alpha_{\text{SSC2(kg)}}+\varepsilon_{i}$$

where k was the breed origin of the paternal SSC2 segment inherited by progeny *i *and g the paternal haplotype transmitted in the candidate region (g = 1,2,3).

For both backcross and F2 pedigrees, significant α_SSC2(kg) _effects (p-value < 0.05) were retained as indicating a QTL detection on SSC2 in the g sub-group of the k group considered. The interaction between the QTL on SSC2 and a second locus in the genome was validated when the effect of the QTL was significant (test p-value < 0.05) in at least one of the groups of sires and not consistent across the (sub-)groups.

## Results

### Sire chromosomes

The backcross population was designed to dissect the region between 7 and 70 cM on SSC2. The five progeny-tested sires were all homozygous by descent for the MS haplotype in the first six centimorgans of SSC2 (Figure 1), the haplotype originating from one founder MS chromosome of the F2 design. Depending on the sire, different contrasts between LW and MS haplotypes were obtained between 7 and 70 cM. It is worthwhile to notice that the Meishan haplotypes were Identical By Descent (IBD) also in this chromosomal region. The LW haplotype origins were more diverse, because different LW animals were introduced during the construction of the pedigree. Among the five progeny-tested sires, three different LW origins were identified. Sires 072866, 072868, 084026 and 085830 inherited the same maternal haplotype, and sire 084024 inherited the alternative maternal chromosome (Figure 1). All of these sires inherited a paternal recombinant chromosome with recombination points between 20 and 35 cM.

### QTL detection on SSC2

Table 1 gives the QTL mapping results for the progeny-tested sires. Two groups of sires were distinguishable based on these results. Three sires (072866, 072868 and 084024) did not segregate for any fatness QTL in the region of interest whereas the two others (084026 and 085830) exhibited consistently significant QTL underlying backfat thickness. QTL effects indicated an increase in fatness attributable to the MS haplotype as compared to the LW haplotypes. The results indicate significant QTL segregation in the regions of heterozygosity LW/MS for these sires (Figure 1). Based on the significant results of sires 084026 and 085830, the QTL underlying fatness traits would be expected to segregate between 27 and 67 cM. Alternatively, the lack of QTL detected within 072866, 072868 and 084024 sires would indicate that the QTL should be localised in the homozygous LW/LW region of these sires. As shown on Figure 1, there was no overlap between the different intervals deduced from the progeny testing results.

**Table 1:** Significant QTL results for fatness traits on SSC2

	Sire 072866		Sire 072868		Sire 084024				Sire 084026					Sire 085830		
					
									QTL results					QTL results		
										
	Nb off.	QTL results^a^	Nb off.	QTL results	Nb off.	QTL results	Nb off.	Max LRT	Pos (cM)	QTL effect	Significance.	Nb off.	Max LRT	Pos (cM)	QTL effect	Significance.
UBFr 120 (mm)	106	-	119	-	98	-	101	8.4	70	-0.44	*	85	9.2	62	-0.47	*
UBFb 120 (mm)	106	-	119	-	98	-	101	9.8	61	-0.45	*	85	9.5	30	-0.38	*
UBFn 120 (mm)	106	-	119	-	98	-	101	8.3	71	-0.58	*	85	9.8	62	-0.65	*
UBFm 120 (mm)	106	-	119	-	98	-	101	11.2	70	-0.49	**	85	9.8	62	-0.46	*
UBFr 140 (mm)	105	-	119	-	98	-	98	-	-	-	-	84	6.9	62	-0.47	*
UBFb 140	105	-	119	-	98	-	98	-	-	-	-	84	7.7	62	-0.57	*
UBFn 140 (mm)	105	-	119	-	98	-	98	10.6	66	-0.67	**	84	9.3	62	-0.75	*
UBFm 140 (mm)	105	-	119	-	98	-	98	6.7	67	-0.44	+	84	9.6	62	-0.59	*
G2 (mm)	74	-	100	-	97	-	94	17.4	67	-1.11	**	72	7.4	31	-0.88	+
M2 (mm)	74	-	100	-	97	-	94	7.1	58	1.39	+	72	-	-	-	-
M6 (mm)	74	-	100	-	97	-	94	7.6	77	1.10	*	72	-	-	-	-
LMC (%)	74	-	100	-	97	-	94	22.7	68	0.98	**	72	-	-	-	-
BFrump (mm)	84	-	102	-	97	-	94	-	-	-	-	72	-	-	-	-
BFback (mm)	84	-	102	-	97	-	94	-	-	-	-	72	-	-	-	-
BFneck (mm)	84	-	102	-	97	-	93	-	-	-	-	72	12	32	-1.67	**
BFmean (mm)	84	-	102	-	97	-	93	-	-	-	-	72	-	-	-	-

QTL effects are given as (maternal) - (paternal) allele effects. +, *, ** indicate the 10%, 5% and 1% significance levels respectively.

UBFr = live ultrasonic backfat thickness measured at the level of the rump; UBFb = live ultrasonic backfat thickness measured at the level of the back; UBFn = live ultrasonic backfat thickness measured at the level of the neck; UBFm = live ultrasonic backfat thickness mean at 120 or 140 days of age. LMC = carcass lean meat content; BFrump = carcass backfat thickness measured at the level of the rump; BFback = carcass backfat thickness measured at the level of the back,; BFneck = carcass backfat thickness measured at the level of the neck; BFmean = average carcass backfat thickness.

### Detection of epistatic interactions

The QTL analyses gave discordant results regarding the localization interval. As shown in table 1 and Figure 2, the most likely position of the SSC2 QTL detected in sires 084026 and 085830 is around 60-70 cM. In this region, sires 072866, 072868, 084026 and 085830 exhibited exactly the same pair of identical by descent chromosomes, and thus carried the same QTL alleles. However, only two of them were validated for the segregation of a fatness QTL, with an effect size of about 0.5 mm on ultrasonic backfat. Family sizes were large enough (N>75 for carcass traits, N>85 for in vivo traits) for all sires to detect this QTL effect with certainty when it segregated. The hypothesis of epistasis was thus considered to explain these discrepancies. For a region to be considered to be candidate as the interacting region, at least two haplotypes should segregate in the four sire families, one enabling the detection of the QTL within sires 084026 and 085830 families, and another masking the segregation of the SSC2 QTL in sires 072866 and 072868 families.

**Figure 3:**
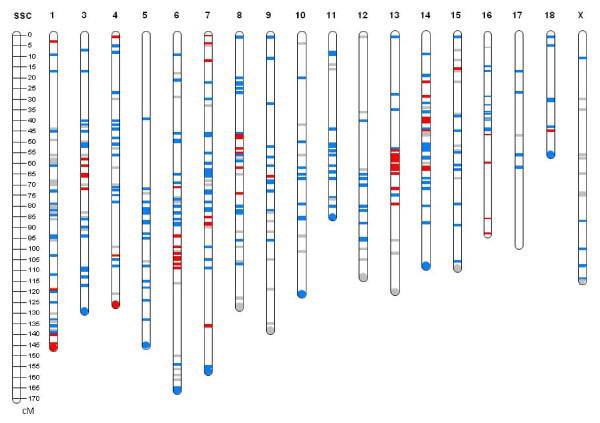
**Genome scan for regions candidate for epistatic interactions with SSC2 in the 072866, 072866, 084026 and 085830 sires**. Each line represents a microsatellite. When two sires did not belong to the same familial group and had the same genotypes, the marker was excluded. In red are the markers retained, in blue are the excluded markers and in grey are the non-informative markers: all the sires were homozygous for the same allele.

To identify these candidate regions, a genome scan was performed on the four sires using a panel of 578 microsatellites distributed throughout the genome (Figure 3). The criteria chosen to select candidate regions were: (1) When two fathers did not belong to the same family group and had the same genotypes then the marker was excluded.(2) A region was selected as candidate when several adjacent markers were retained. The genotypes in the two groups of sires (072866/072868 *versus *084026/085830) were thus compared. Finally, seven different candidate regions, on SSC1 (140-145 cM), SSC3 (55-75 cM), SSC6 (90-120 cM), SSC8 (50-60 cM), SSC13 (55-75 cM), SSC14 (20-35 cM) and SSC16 (45-90 cM) were selected (Figure 3).

One microsatellite (two for SSC13) was retained to mark each region to be genotyped on the four sire progeny. An interval mapping strategy was applied for the two groups of sires independently (072866/072868 or 084026/085830), using the model (1) where the interaction between the microsatellite alleles and the effect of every putative position of the SSC2 QTL within the 40-80 cM region was tested. This analysis was applied only to ultrasonic backfat thickness measured at the level of the neck at 140 days of age, which showed the highest significance of the SSC2 QTL in the 084026 and 085830 families (Figure 2). Only the results obtained at the SW395 position (66 cM), microsatellite genotyped in all families closest to the most likely position of the SSC2 QTL, are presented (table 2).

**Table 2:** Interaction analyses results

		Sires 072866 and 072868			Sires 084026 and 085830		
			
Tested marker	Allele	Nb off.	Effect	P-val	Nb off.	Effect	P-val
	1	69	-0.51	0.308	58	-1.45	0.009
SSC1 (SW2512)	2	81	-0.27	0.544	-	-	-
	3	-	-	-	67	-1.27	0.010

	1	85	-1.49	**0.001**	87	-1.86	<0.0001
SSC3 (SW1436)	2	93	0.37	0.372	-	-	-
	3	-	-	-	70	0.60	0.242

	1	62	-1.07	**0.050**	67	-1.49	0.007
SSC6 (SW1055)	2	55	-0.19	0.719	-	-	-
	3	-	-	-	91	-1.27	0.005

	1	64	-0.77	0.186	-	-	-
SSC8 (SW205)	2	77	-0.34	0.414	57	-0.74	0.147
	3	-	-	-	65	-1.57	0.003

	1	69	-0.54	0.286	73	-1.69	0.001
SSC13 (SW207)	2	66	0.32	0.540	-	-	-
	3	-	-	-	67	-1.01	0.046

	1	93	-0.95	**0.025**	82	-1.60	0.001
SSC13 (SW1550)	2	80	0.01	0.980	-	-	-
	3	-	-	-	67	-1.10	0.024

	1	83	0.09	0.833	45	-1.28	0.024
	2	85	-0.66	0.164	-	-	-
SSC14 (SW245)	3	-	-	-	76	-1.23	0.009
	4	-	-	-	36	-1.67	0.059

	1	74	-0.56	0.220	72	-1.39	0.009
SSC16 (MCSeq5008)	2	70	-0.48	0.350	-	-	-
	3	-	-	-	64	-1.31	0.012

Interactions were tested between eight microsatellites (representing eight candidate regions) at position 66 cM on SSC2. QTL effects α_SSC2(kg) _given as (MS - LW) and the associated p-values are shown. In bold are the significant p-values obtained for sires 072866 and 072868 for which no QTL was detected on SSC2

Model (1) was applied to the two groups of sires (072866/072868 *versus *084026/085830). The test p-values were always significant when the sires 084026 and 085830 were analysed (from 0.0034 to 0.0003), and were never significant (the smallest p-value being 0.10 when interaction was tested with SW1550 on SSC13) when sires 072866 and 072868 were analysed.

On SSC1, three different alleles were observed, allele 1 being present in the two groups of sires but the SSC2 QTL effect being detected only in sire 084026 and 085830 families. A similar pattern was observed on SSC14, SSC13 (SW207), and SSC16. As a result, regions on SSC1, around SW207 on SSC13, SSC14 and SSC16 were not retained as candidate regions because a common allele gave different results in the two groups of sires.

For the SSC3 candidate region, the SSC2 QTL effect (at 66 cM on SSC2) was detected in the two groups of sires, but only when the progeny inherited allele 1 at the tested microsatellite. For SSC8, the only allele enabling the detection of the SSC2 QTL segregated in the progeny of sires 084026 and 085830. These two regions on SSC3 and SSC8, were conserved as candidate epistatic regions, as significant interactions were obtained with only one allele of the tested microsatellite, segregating in one (SW205 on SSC8) or the two groups of sires (SW1436 on SSC3).

On SSC6 and for SW1550 on SSC13, all the alleles except for one were associated with significant detection of the SSC2 QTL. On each of these two microsatellites, the allele for which the SSC2 QTL could not be detected segregated only in the sires of the 072866 and 072868 families. Thus, SSC6 around SW1550 on SSC13 were considered as candidate regions for interaction with the SSC2 QTL.

**Figure 4:**
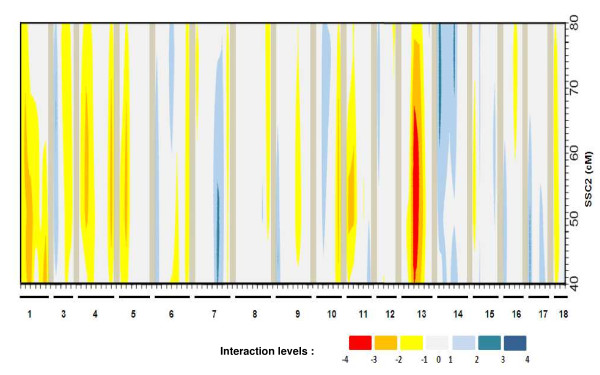
**Contrasts between p-values (log_10 _scale) obtained for interval mapping tests within progeny with LW paternal alleles and within progeny with MS paternal alleles on the PorQTL pedigree**. Interval mapping tests were applied on SSC2 (y-axis, 40-70 cM region) with all available positions in the genome scan as candidate for interaction (x-axis, from SSC1 to SSC18 (SSC2 being excluded)). A blue (orange) spot indicates that the SSC2 QTL is more likely to be detected in progeny that inherited the Large White (Meishan) allele paternally at the candidate position for interaction. Extreme colors indicate the highest p-value differences, i.e. the strongest candidates for interaction with the SSC2 QTL.

In order to reduce the number of candidate regions, a complementary genome scan was carried out using the F2 PorQTL design. The F2 animals were grouped based on the breed origin (LW or MS) of their paternal allele. All the microsatellites available from the original genome scan of the population were tested by interval mapping as previously described for interactions within the 40-70 cM region on SSC2. A region on SSC13 covered with three microsatellites (S0222, SW225 and SW38) showed the highest significance for an interaction with the SSC2 region (Figure 4): the SSC2 QTL was detected only in F2 animals which inherited the LW haplotype from their sire on SSC13. The SSC13 region spanning 30 cM from SW225 to SW38, containing the SW1550 microsatellite used in the backcross population, was therefore considered to be the strongest candidate region for the interaction with the SSC2 QTL in both F2 and backcross pedigrees.

**Figure 5:**
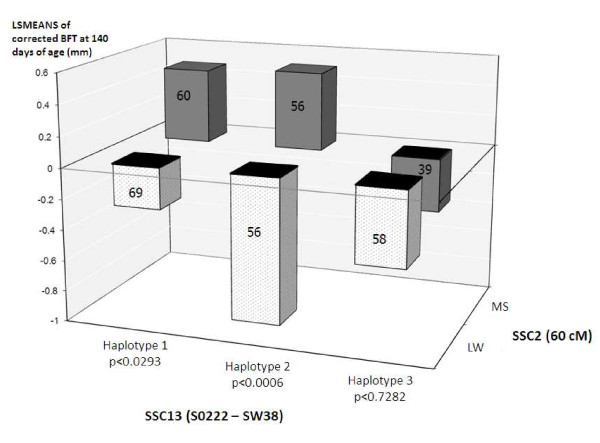
**Least-Squares MEANS of corrected values of live BFT measured at 140 days of age for progeny of sires 072866, 072868, 084026 and 085830**. LSMEANS were computed for each combination of SSC13 paternal haplotype designed from S0222 to SW38 and paternal SSC2 allele breed origin at 66 cM. The number of animals per group is indicated on each bar. P-values, below the haplotype names, refer to the Student's tests applied to the LSMEANS differences obtained within each SSC13 haplotype, the p-value of the test being less than 0.0006.

The three microsatellites of the SSC13 region that were significant in the F2 design were genotyped on the backcross animals. First, sire haplotypes were constructed from the familial segregation information. Three different haplotypes were segregating in 072866, 072868, 084024 and 085830 sires. Sire 084026 was heterozygous for haplotypes 1 and 2, sire 085830 was homozygous for haplotype 2, and sires 072866 and 072868 were heterozygous for haplotype 1 and haplotype 3. Second, the interaction of these haplotypes with the SSC2 region was tested. Progeny which inherited the paternal haplotype 1 or haplotype 2 showed significantly different phenotypic values according to the breed origin of their paternal haplotype at 66 cM on SSC2 (Figure 5). By contrast, progeny that inherited the paternal SSC13 haplotype 3 showed no differences in phenotypic values based on their paternal haplotype on SSC2.

## Discussion

The aims of this study were to confirm and fine-map a QTL underlying fatness traits segregating around 30-70 cM downstream of the *IGF2 *gene through the production of animals dedicated to this project. Progeny testing of 084026 and 085830 sires confirmed that a QTL affecting fatness was localized around 60-70 cM on SSC2. Surprisingly, the two additional full sibs 072866 and 072868, which shared the same identical-by-descent haplotypes in this region, showed no evidence for this QTL. To explain this discrepancy, eight regions (localised on seven different chromosomes) were retained and tested for interactions with SSC2. As concerns the two regions on SSC3 and SSC8, significant results were obtained with only one allele of the tested microsatellite. In these regions, interaction would imply that only one allele enabled the detection of a significant QTL effect on SSC2. In these cases, the power of the design to detect the SSC2 QTL would be influenced by the proportion of offspring carrying these alleles. Alternatively, for the regions selected on SSC6 and SSC13 (SW1550), the SSC2 QTL was detected with all alleles except one segregating within the 072866/072868 sires' families. This particular allele on SSC6 or SSC13, inhibited the segregation at SSC2 QTL. For these two candidate regions, this suggests that one allele inhibits the MS QTL allele of SSC2. An additional argument in favour of the SSC13 region was provided by the analysis of the F2 PorQTL design, in which this region was the strongest candidate for interaction with SSC2 in a whole genome scan. Here, the QTL was detected on SSC2 when the progeny inherited a LW allele from their sire in the SSC13 region. SW207 is in this interacting region, and the non-significant result obtained (table 2) may be due to the low number of progeny to which a paternal allele could be attributed with certainty, as compared to SW1550.

Number of studies already aimed at identifying QTL interactions in pig [20-27], and many different pairs of interacting QTL were described underlying various traits including fatness. By comparing our results to the ones from these seven extant papers, no identical epistatic pair of regions may be found in common. This could be attributable to the different traits analysed, the different breeds involved in the pedigrees and to the power of the analyses. In all these studies, few pairs of interacting QTL were detected in contrast to studies carried out in mice where all detected QTL underlying adiposity were involved in epistatic interactions [28]. A comparison of the regions detected in the pig in this study and orthologous regions in the mouse indicated no overlap in known epistatic genes. This indicates that the interaction described in this study between SSC2 and SCC13 is novel in both pigs and mice.

Most of the studies in search of epistasis were based on the Line-Cross model which assumes that founder lines are fixed for alternative QTL alleles for the trait of interest, so that each progeny-tested sire is heterozygous for each interacting QTL. This classifies the offspring in sixteen different classes, reduced to nine classes by merging the heterozygous individuals at each interacting locus, as illustrated in [24]. This assumption can be easily verified in mouse experiments using inbred lines but it is much more difficult to demonstrate in pigs or other livestock animals. For example, we already know, through the analysis of the *IGF2*-intron3-G3072A mutation, that the assumption of fixed alleles is not always confirmed [2]., Because the QTL alleles fixation cannot be considered as a rule, therefore it is very likely that animals carrying different QTL alleles can belong to the same "breed-based" class. This confusion between QTL alleles and breeds could partly explain why few interacting pairs of QTL are detected compared to mice. Additionally, it has been shown that departure from the Line Cross hypothesis decreases the power to detect epistasis [29]. The Line Cross model remains highly used for epistatic interaction as it allows keeping group sizes large enough to afford sufficient statistical power. For this reason, it was also used in this study for the analysis of the F2 animals, considering only the segregation of the paternal alleles. However, finer interaction analyses were performed with the BC families, as it obviated any confusion between the breeds and the QTL alleles. The four sires analysed were full sibs and were IBD for both the paternal and maternal haplotypes in the 40-66 cM region of SSC2. Therefore, by construction, they all shared the same alleles (Q and q alleles) at the SSC2 QTL. This binary situation could however only be asserted in this SSC2 region, and not for the rest of the genome. Nevertheless, because the four sires analysed for epistasis were full-sibs, only a few different alleles were segregating and, for a pool of two sires, interactions have been tested with a maximum of three different alleles. This advantageous situation enabled us to work out of the Line Cross assumption and to determine that among the three possible haplotypes segregating in the SSC13 region only one inhibited the segregation at the SSC2 QTL.

### QTL detection and localization

In this analysis, a QTL underlying fatness traits was detected around 60-70 cM, with Meishan alleles increasing fatness. Based on the recombination points, the QTL region spanned 30 cM and was delimited by the recombination point on the paternal allele of 072868 (at 37 cM) and by the one at the end of the Meishan haplotype on sire 085830 (at 67 cM). This study confirmed previous analyses of SSC2 based on Meishan and European White breeds such as Large White and Landrace, where Chinese alleles were always associated with a higher fatness as compared to European White breeds alleles [6,7,30]. However, QTL intervals were different according to the trait with a region from 0 to 30 cM underlying backfat thickness measured on living animals [6] and a 43 cM region from 40 to 83 cM underlying backfat thickness measured on carcass [7,30]. In a Meishan-White composite resource population, a QTL underlying backfat thickness measured at 14 weeks of age was detected around 74 cM [31], which is consistent with the most likely positions we detected here. In crosses between Pietrain and Wild boar or Meishan, QTL underlying carcass fatness traits were mainly detected between 55 and 75 cM on SSC2 and not in the first centimorgans, even if the Pietrain breed is nearly fixed for the *IGF2*-intron3-3072A allele and Wild Boars and Meishan are supposed to be fixed for the alternate allele [32]. When the *IGF2*-intron3-G3072A mutation was taken into account in the analysis of Meishan × European White breed crosses, the segregation of a QTL underlying BFT around 40-50 cM was observed [15], and the segregation of QTL affecting fatness traits between 40 cM and 60 cM was also reported by Lee et al in a Wild Boar × Meishan pedigree were all founders animals were G/G for the *IGF2*-intron3-G3072A mutation [11]. In our study also, all the progeny-tested sires were homozygous G/G for the *IGF2*-intron3-G3072A mutation so variation of the studied traits would not be due to this polymorphism.

### Fine-mapping through the production of additional animals

The backcross design was set up to confirm and fine-map a QTL underlying fatness traits, segregating around 30-70 cM on SSC2. This marker-assisted backcrossing method already gave encouraging results in crosses involving European breeds mated with Meishan or Wild Boars [10,12,13] and a highly significant result with the decrease from 70 to 3.3 cM of the FAT1 QTL interval on SSC4 [33]. If in our case this design was not directly succesfull in decreasing the QTL confidence interval, it has allowed interactions between the SSC2 QTL and a locus mapped on SSC13 to be detected. Despite being a long and expensive method requiring a lot of animals, marker-assisted backcrossing remains a powerful method to finely dissect segregating QTLs in livestock.

## Conclusion

The marker-assisted backcrossing method presented in this study ended first with the confirmation of a fatness QTL between 37 and 67 cM on SSC2 and then with the highlighting of interactions between this QTL and at least another region on SSC13. As far as we know, it is the first time that such a design has enabled the detection of epistasis. In addition, by considering only different haplotypes whatever the original breed, no assumption of line-cross model was used. This study reappraises the marker-assisted backcrossing strategy as not only an efficient method to fine-map QTL but also to understand the mode of their segregation. When such interactions are described, additional steps are needed to improve the mapping accuracy of the locus, which can delay the fine-mapping of the QTL. Therefore, the marker-assisted backcrossing design provides valuable information which, however, has to be balanced with the time that it requires for fine-mapping.

## Competing interests

The authors declare that they have no competing interests.

## Authors' contributions

FT and KF carried out the genotyping of the backcross families, KF developed microsatellite markers, NI carried out the genome wide genotyping for the detection of epistatic regions. FT performed the epistatic analysis and, with MPS, the QTL analyses. HG supervised the statistical analyses. YB supervised the performance testing, from animal production to biological sampling. JPB and DM were co-responsible of the INRA QTL design. JR proposed the idea and had the responsibility of the follow-up of the experiment. All the authors read and approved the final manuscript.
